# Parity status and the relationship between antenatal rubella serology with obstetric outcome

**DOI:** 10.1038/s41598-022-05376-8

**Published:** 2022-01-24

**Authors:** Terence T. Lao, Shuk Yi Annie Hui, Daljit S. Sahota

**Affiliations:** Department of Obstetrics and Gynaecology, The Chinese University of Hong Kong, Prince of Wales Hospital, Shatin, NT, Hong Kong SAR People’s Republic of China

**Keywords:** Diseases, Health care, Risk factors

## Abstract

Routine antenatal rubella serological testing is adopted in many countries. In a population covered by universal childhood rubella immunization for four decades, we have observed an association between pre-eclampsia with maternal rubella seronegativity among multiparous gravidae. This retrospective cohort study was further performed to elucidate the interaction between parity status and rubella seronegativity on obstetric outcome in singleton pregnancies carried to ≥ 24 weeks gestation managed from 1997 to 2019, with the data retrieved from a computerized database used for annual statistics and auditing. Of the 133,926 singleton pregnancies eligible for the study, the 13,320 (9.9%) rubella seronegative gravidae had higher mean booking weight and body mass index (BMI), but shorter height, and higher incidence of advanced age (≥ 35 years), high BMI, short stature, and lower incidence of nulliparas. Univariate analysis showed that adverse obstetric outcomes were more frequently found among the multiparas. On multivariate analysis, there was increased postdated (> 41 weeks) pregnancy irrespective of parity status, while nulliparas had reduced gestational hypertension (aRR 0.714, 95% CI 0.567–0.899) and gestational diabetes (aRR 0.850, 95% CI 0.762–0.950), and multiparas had increased pre-eclampsia (aRR 1.261, 95% CI 1.005–1.582), neonatal death (aRR 2.796, 95% CI 1.243–6.291), and perinatal death (aRR 2.123, 95% CI 1.257–3.587). In conclusion, in a population covered by universal childhood rubella immunization, antenatal rubella seronegativity is associated with increased pre-eclampsia and perinatal loss only in multiparas, suggesting that the rubella seronegativity in these women served as proxy for some form of altered immune response which increases adverse pregnancy outcome.

## Introduction

With the introduction of the live-attenuated rubella vaccine to prevent congenital rubella syndrome^1^, the rate of rubella sero-negativity at antenatal screening has been reduced to < 5% in developed countries such as Finland^2^, Australia^3^, USA^4^ and Taiwan^5^. Nevertheless, it was also shown that the overall seronegative rate in women aged 15–39 years in European countries which have adopted universal immunization remained variable from 1.4% in the Czech Republic to 13.4% in Belgium^6,7^. While there might be a number of different reasons to explain the variable incidence of rubella sero-negativity in the aforementioned European countries^6,7^, one underlying cause could have been vaccine non-response in some of the seronegative women.

In Hong Kong, rubella immunization was introduced in 1978, which evolved to the current two-dose mumps-measles-rubella (MMR) vaccination regimen at 12 months and 12 years of age from 1996 with 99% coverage of all primary six students, together with catch-up vaccination provided to susceptible women of reproductive age as well as postnatal immunization to seronegative gravidae^8,9^. Despite the rigorous implementation of rubella immunization, antenatal screening revealed that 8.1% of the gravidae were seronegative^10^, a situation similar to the findings in Europe^6,7^. However, whether antenatal rubella sero-negativity within an obstetric population covered by universal childhood immunization has implications on pregnancy outcome remains unclear, owing to the scanty literature on the subject. A case–control study which examined the obstetric outcome by rubella immunity status found no difference in adverse outcome between 285 cases with rubella IgG < 10 IU/ml and no underlying medical conditions with 285 controls^11^. However, the study was probably underpowered, as the incidence of pre-eclampsia (PE) in the cases was 3.5% which was almost 50% higher than the 2.4% in the controls. On the other hand, we have found that rubella seronegative status was associated with increased PE among multiparous but not nulliparous gravidae^12^, suggesting that rubella sero-negativity in our population could be a surrogate for subtle differences in maternal immune status which could predispose towards PE. It is known that maternal tolerance of the foetal-placental unit in a successful pregnancy involves a shift to a distinct Th2 bias with decreased Th1 cytokines that begins from the first trimester to peak in the third trimester^13,14^, and the immunologic status changes to a pro-inflammatory phase in the first trimester, an anti-inflammatory phase in subsequent trimesters, and finally returning to a renewed inflammatory phase at parturition^15^. Indeed, different maternal cytokine profiles were found to associate with pregnancy complications^16^, and whether rubella sero-negativity among vaccinated women could be proxy to some form of underlying differences in maternal immunological status remains an unanswered question. We therefore performed this retrospective cohort study to compare obstetric outcomes between rubella seronegative with seropositive gravidae to determine if rubella immunity status has any bearing on pregnancy outcome in addition to the influence on PE as reported before^12^.

## Materials and methods

Our hospital is a public regional tertiary referral centre catering for a population of 1.7 million, currently with an annual delivery rate of around 7000 which consisted almost entirely of public patients. Routine antenatal screening for rubella IgG antibody titre by EIA (Axsym, Abbott) is performed at the first antenatal attendance. The test is performed by the accredited central laboratory under the Department of Health (DH) if the antenatal booking visit occurs at the maternal and child health centres (MCHC), and in the accredited hospital pathology laboratory if the booking visit occurs at the hospital. Women with a titre of ≥ 10 IU/ml are reported as immune, while seronegative women with antibody titre < 10 IU/ml are reported as non-immune, but the actual titre is not provided in the report. Women seronegative for rubella are referred after delivery for rubella vaccination at the MCHC where their babies will also receive the rest of the immunization program for infancy. However, details of postpartum rubella vaccination were not available as we could not access the postnatal management and vaccination data from DH statistics. Antenatal screening results, clinical information, and medical intervention, and obstetric outcome, are all entered into our computerized patient record and the database is used for both clinical management and generation of annual statistics for our Hospital Authority.

In this retrospective study, we categorized gravidae carrying a singleton pregnancy to beyond 24 weeks gestation and delivering in our hospital between 1997 and 2019 by their rubella serology status for comparison of maternal characteristics and pregnancy outcome. Maternal characteristics included established obstetric risk factors and medical history^17^, and specific obstetric risk factors included nulliparous status and advanced (≥ 35 years) age^18,19^, body mass index (BMI) at booking and incidence of high (> 25 kg/m^2^) BMI, height and incidence of short stature defined as height at or less than the 25th percentile of our obstetric population which has influence on the occurrence of pregnancy hypertensive disorders^20^, history of abortion which, inclusive of both spontaneous and induced as well as just one or recurrent abortions, is an usually overlooked risk factor which impacts on subsequent pregnancy outcome including the development of pregnancy hypertensive disorders^21–26^, and carried a male foetus^27^. Pregnancy outcome analysed included gestational hypertension (GH), PE, gestational diabetes (GDM), placenta praevia, placental abruption, preterm birth (< 37 weeks), post-dated pregnancy (carried to beyond 41 weeks gestation), labour induction, caesarean section (CS), manual removal of placenta (MROP), postpartum haemorrhage (PPH), infant gender, infant macrosomia (birthweight ≥ 4000 g), low birthweight infants (birthweight < 2500 g), Apgar score at 5th minute < 7 as an indication of asphyxia, and total perinatal death, and stillbirth and neonatal death. The diagnosis of PE was made according to the International Society for the Study of Hypertension in Pregnancy criteria^28^. As pregnancy-induced hypertension in the literature sometimes included both hypertension with and without proteinuria, we avoided any confusion by separating women with hypertension diagnosed after 20 weeks gestation without proteinuria (including those with unknown blood pressure before 20 weeks of gestation) as GH; while PE referred to hypertension with significant proteinuria, including women with PE superimposed on pre-existing hypertension, so that these groups which are distinct entities^29^ did not overlap since the diagnosis was finalised at delivery. With the exception of a handful of emergency transfers, all of whom were before term, practically all the pregnancies had the gestational age confirmed by early foetal ultrasonography performed to establish the gestational age in gravidae with uncertain dates, or irregular or prolonged menstrual cycles, and for screening of fetal structural anomalies and chromosomal abnormalities such as trisomy 21, so that the gestational age captured in the obstetric database were confirmed, and post-dated pregnancies were defined as pregnancies carried to beyond 41 completed weeks. Owing to a rigorously organised foetal screening programme, pregnancies carrying foetuses with lethal or significant congenital and chromosomal anomalies were invariably detected before delivery, mostly before foetal viability (24 completed weeks of gestation). As therapeutic termination of pregnancy before 24 weeks gestation was and still is legal in Hong Kong, almost all of these pregnancies were terminated before foetal viability and therefore not captured in the obstetric database, which therefore does not include details of foetal anomalies. Further analysis was performed stratified by maternal parity status owing to the known influence of parity status on pregnancy outcome^30^. Statistical analysis was performed using t test and chi square test and calculation of relative risk (RR) with 95% confidence intervals (CI). Finally, the independent association between rubella sero-negativity with the examined pregnancy outcomes was determined by multiple logistic regression analysis considering the relevant risk factors identified, with the results expressed in adjusted relative risk (aRR) and 95% confidence intervals (CI). Statistics analysis was performed using a commercial statistical package (PASW Statistics 26.0, SPSS Inc., Chicago, Il).

The study was approved by the Institutional Review Board (Joint Chinese University of Hong Kong–New Territories East Cluster Clinical Research Ethics Committee, Reference Number CRE-2017.442), which has waived informed consent from the study subjects eligible for the study as requested in the submission for ethical approval by the authors (SYAH and DSS) because the study utilized an anonymized database spanning 23 years. All methods were performed in accordance with the relevant guidelines and regulations.

### Ethics approval

The study has received ethics approval from the Institutional Body ((Joint Chinese University of Hong Kong—New Territories East Cluster Clinical Research Ethics Committee, Reference Number CRE-2017.442).

### Consent for publication

Given by all authors.

## Results

There were 133,926 cases in the database which had satisfied the inclusion criteria, and rubella sero-negativity was found in 13,320 (9.9%) cases. Although there was no significant difference in the mean maternal age (Table 1), rubella seronegative gravidae had significantly higher mean booking weight and BMI but shorter height, and had higher incidence of advanced age (RR 1.162, 95% CI 1.128–1.197), high BMI (RR 1.244, 95% CI 1.208–1.280), short stature (RR 1.120, 95% CI 1.089–1.152), but lower incidence of nulliparous women (RR 0.919, 95% CI 0.902–0.937). The mean gestational age and birthweight were only very slightly but still significantly higher, but there was no difference in the incidence of male infants.

**Table 1:** Maternal and infant characteristics between gravidae seronegative versus seropositive for rubella immunity.

	Rubella status		*p*
	Seronegative	Seropositive	
Number (% of total)	13,320 (9.9)	120,606 (90.1)	*–*
Age (years)	30.6 ± 5.8	30.6 ± 5.4	0.943
Age ≥ 35 years (%)*	26.9	23.1	< 0.001
Height (cm)	157.4 ± 5.5	158.1 ± 5.5	< 0.001
Height ≤ 154 cm (%)*	29.4	26.2	< 0.001
Booking weight (kg)	57.9 ± 9.5	56.9 ± 9.3	< 0.001
Booking BMI (kg/m^2^)	23.4 ± 3.6	22.8 ± 3.5	< 0.001
BMI ≥ 25 kg/m^2^ (%)*	28.1	22.6	< 0.001
Nulliparous gravidae (%)*	47.8	52.0	< 0.001
Gestational age (wks)	38.9 ± 1.9	38.8 ± 1.8	0.016
Birthweight (g)	3182 ± 491	3152 ± 479	< 0.001
Male infant (%)*	52.2	52.0	0.692

*BMI* body mass index.

Results expressed in mean ± SD and compared with the t test, or in % as indicated* and compared with the chi square test.

When the history and pregnancy outcome were compared, the rubella seronegative gravidae had significantly higher incidence of abortion history, previous CS among the multiparas, obstetric complications overall, PE, post-dated pregnancy, CS, MROP, macrosomic infants, and neonatal death (Table 2). On the other hand, they had significantly lower incidence of past medical history and pre-existing conditions overall, GH, labour induction, and PPH. The incidence of most of the individual medical conditions were very low so that only the important ones which are known risk factors for adverse pregnancy outcomes including chronic hypertension, pre-gestational diabetes, and autoimmune conditions, were further analysed, but no significant difference could be found between rubella seronegative and seropositive gravidae.

**Table 2:** Obstetric outcome between rubella seronegative (n = 13,320) versus seropositive (n = 120,606) gravidae.

	Rubella status		*p*	RR (95% CI)
	Seronegative	Seropositive		
Medical history	**10.6**	**12.7**	** < 0.001**	**0.837 (0.795–0.881)**
Chronic hypertension	0.4	0.4	0.609	0.933 (0.744–1.219)
Pre-gestational diabetes	0.4	0.3	0.096	1.269 (0.962–1.674)
Autoimmune disorders	0.4	0.3	0.620	1.070 (0.819–1.399)
Abortion history	**49.1**	**43.9**	** < 0.001**	**1.119 (1.098–1.140)**
Previous cesarean delivery^a^	**18.8**	**16.9**	** < 0.001**	**1.109 (1.047–1.174)**
Obstetric complications	**21.7**	**20.4**	**0.021**	**1.061 (1.009–1.115)**
Gestational hypertension	**1.1**	**1.4**	**0.008**	**0.794 (0.670–0.941)**
Pre-eclampsia	**1.7**	**1.4**	**0.018**	**1.185 (1.030–1.362)**
Gestational diabetes	8.5	8.1	0.171	1.042 (0.982–1.106)
Placental abruption	1.1	1.1	0.307	1.091 (0.923–1.289)
Placenta praevia	1.0	1.0	0.689	1.013 (0.846–1.213)
Preterm birth < 37 weeks	6.6	6.4	0.446	1.027 (0.960–1.098)
Post-dated pregnancy	**1.6**	**0.9**	** < 0.001**	**1.669 (1.442–1.930)**
Labour induction	**22.0**	**22.9**	**0.017**	**0.960 (0.928–0.993)**
Instrumental vaginal delivery	**12.3**	**13.1**	**0.036**	**0.938 (0.884–0.996)**
Caesarean section	**23.3**	**22.0**	**0.001**	**1.059 (1.025–1.094)**
Manual removal of placenta	**4.1**	**3.6**	**0.002**	**1.145 (1.049–1.248)**
Postpartum haemorrhage	**3.1**	**3.4**	0.088	0.918 (0.831–1.013)
Infant ≥ 4000 g	**3.7**	**2.9**	** < 0.001**	**1.286 (1.172–1.411)**
Infant < 2500 g	6.4	6.6	0.301	0.965 (0.901–1.033)
Male infant	**52.5**	**52.0**	0.358	1.008 (0.991–1.025)
Apgar score < 7 at 5th min	0.6	0.5	0.340	1.124 (0.884–1.434)
Perinatal mortality	0.5	0.4	0.077	1.273 (0.974–1.663)
Stillbirth	0.3	0.2	0.436	1.143 (0.816–1.603)
Neonatal death	**0.2**	**0.1**	**0.045**	**1.566 (1.006–2.437)**

Significant differences shown in bold.

Results in %, analysis with chi square test and relative risk (RR) and 95% confidence intervals (CI).

^a^Denotes analysis among multiparous gravidae.

Owing to the significant difference between the two groups in the incidence of multiparous gravidae, past history and pregnancy outcome were further analysed stratified by parity status. Among nulliparous gravidae (Table 3), there was significantly higher incidence of abortion history, obstetric complications overall, post-dated pregnancy, and macrosomic infants, whereas there was lower incidence of medical history, GH, and labour induction.

**Table 3:** Obstetric outcome between rubella seronegative (n = 6363) versus seropositive (n = 62,669) nulliparous gravidae.

	Rubella status		*p*	RR (95% CI)
	Seronegative	Seropositive		
Medical history (%)	**10.9**	**12.9**	** < 0.001**	**0.847 (0.787–0.911)**
Abortion history (%)	**40.4**	**37.5**	** < 0.001**	**1.076 (1.043–1.111)**
Obstetric complications (%)	**20.7**	**18.8**	**0.015**	**1.096 (1.018–1.180)**
Gestational hypertension	**1.3**	**1.7**	**0.023**	**0.772 (0.617–0.966)**
Pre-eclampsia	2.0	1.8	0.268	1.109 (0.924–1.332)
Gestational diabetes	6.2	6.7	0.198	0.937 (0.848–1.035)
Placental abruption	1.2	1.1	0.390	1.107 (0.878–1.395)
Placenta praevia	0.8	0.8	0.645	0.934 (0.700–1.247)
Preterm birth < 37 weeks	6.5	6.6	0.699	0.981 (0.889–1.082)
Post-dated pregnancy	**1.7**	**0.9**	** < 0.001**	**1.900 (1.550–2.329)**
Labour induction	**27.3**	**28.6**	**0.037**	**0.957 (0.917–0.998)**
Instrumental vaginal delivery	20.6	20.6	0.971	1.001 (0.939–1.068)
Caesarean section	23.6	22.9	0.216	1.030 (0.983–1.079)
Manual removal of placenta	**4.9**	**3.8**	** < 0.001**	**1.270 (1.132–1.426)**
Postpartum haemorrhage	3.2	3.6	0.080	0.881 (0.764–1.015)
Infant ≥ 4000 g	**2.7**	**2.0**	** < 0.001**	**1.344 (1.147–1.574)**
Infant < 2500 g	7.2	7.7	0.150	0.934 (0.852–1.025)
Male infant	51.1	51.7	0.346	0.988 (0.963–1.013)
Apgar score < 7 at 5th min	0.5	0.6	0.532	0.893 (0.626–1.274)
Perinatal mortality	0.4	0.4	0.491	0.861 (0.563–1.318)
Stillbirth	0.2	0.3	0.261	0.733 (0.426–1.262)
Neonatal death	0.1	0.1	0.635	1.182 (0.592–2.359)

Significant differences shown in bold.

Results in %, analysis with chi square test and relative risk (RR) and 95% confidence intervals (CI).

Among multiparous gravidae (Table 4), there was lower incidence of medical history, but higher incidence of abortion history, PE, GDM, post-dated pregnancy, CS, macrosomic infants, male infants, low fifth minute Apgar score, overall perinatal mortality, stillbirth and neonatal death.

**Table 4:** Obstetric outcome between rubella seronegative (n = 6957) versus seropositive (n = 57,937) multiparous gravidae.

	Rubella status		*p*	RR (95% CI)
	Seronegative	Seropositive		
Medical history (%)	**10.4**	**12.5**	** < 0.001**	**0.830 (0.772–0.892)**
Abortion history (%)	**57.1**	**50.8**	** < 0.001**	**1.124 (1.100–1.149)**
Obstetric complications (%)	22.6	22.1	0.480	1.025 (0.958–1.097)
Gestational hypertension	0.9	1.0	0.273	0.865 (0.667–1.122)
Pre-eclampsia	**1.4**	**1.0**	**0.003**	**1.388 (1.117–1.724)**
Gestational diabetes	**10.5**	**9.7**	**0.034**	**1.082 (1.006–1.164)**
Placental abruption	1.1	1.0	0.498	1.087 (0.854–1.383)
Placenta praevia	1.1	1.1	0.684	1.049 (0.838–1.322)
Preterm birth < 37 weeks	6.7	6.2	0.121	1.077 (0.981–1.183)
Post-dated pregnancy	**1.5**	**1.0**	** < 0.001**	**1.465 (1.188–1.805)**
Labour induction	17.1	16.8	0.483	1.020 (0.965–1.077)
Instrumental vaginal delivery	5.1	5.2	0.694	0.974 (0.852–1.113)
Caesarean section	**23.1**	**22.1**	** < 0.001**	**1.096 (1.047–1.147)**
Manual removal of placenta	3.4	3.4	0.702	1.026 (0.899–1.171)
Postpartum haemorrhage	3.1	3.2	0.586	0.962 (0.838–1.109)
Infant ≥ 4000 g	**4.6**	**3.8**	**0.001**	**1.209 (1.079–1.355)**
Infant < 2500 g	5.6	5.4	0.513	1.035 (0.934–1.146)
Male infant	**53.7**	**52.4**	**0.039**	**1.025 (1.002–1.049)**
Apgar score < 7 at 5th min	**0.6**	**0.4**	**0.021**	**1.472 (1.057–2.050)**
Perinatal mortality	**0.5**	**0.3**	** < 0.001**	**1.851 (1.303–2.628)**
Stillbirth	**0.3**	**0.2**	**0.010**	**1.769 (1.139–2.746)**
Neonatal death	**0.2**	**0.1**	**0.017**	**2.010 (1.122–3.601)**

Significant differences shown in bold.

Results in %, analysis with chi square test and relative risk (95% confidence intervals).

To determine the independent association between rubella seronegative status with the different pregnancy outcomes demonstrated in the univariate analysis, multiple logistic regression analysis was performed (Table 5). In Model One, the relationship with obstetric complications overall, GDM, GH and PE was examined with adjustment for advanced maternal age, high BMI, short stature, male foetus, previous abortion history, and the presence of medical history. For the entire cohort, rubella seronegative status was associated with reduced GH, but increased post-dated pregnancy and neonatal death. When analysis was confined to nulliparous gravidae, rubella seronegative status was associated with reduced GDM and GH, but increased post-dated pregnancy. When analysis was confined to multiparous gravidae, rubella seronegative status was associated with increased PE, post-dated pregnancy, perinatal death and neonatal death. The reasons for the increased perinatal and neonatal deaths in the rubella seronegative group could not be determined because the baby records were set up as separate from those of their mothers (often with their new given names) in the computer system and could not be captured in the obstetric database.

**Table 5:** Multiple logistic regression analysis of the independent association between rubella seronegativity with specific pregnancy outcomes.

	Whole cohort	Nulliparas	Multiparas
Any obstetric complication^a^	1.049 (0.982–1.120)	1.074 (0.977–1.181)	1.020 (0.931–1.116)
Gestational diabetes^a^	0.947 (0.886–1.013)	**0.850 (0.762–0.950)**	1.014 (0.931–1.103)
Gestational hypertension^a^	**0.757 (0.637–0.900)**	**0.714 (0.567–0.899)**	0.818 (0.628–1.066)
Pre-eclampsia^a^	1.142 (0.987–1.321)	1.066 (0.881–1.289)	**1.261 (1.005–1.582)**
Preterm birth^b^	1.007 (0.935–1.085)	0.874 (0.744–1.028)	0.995 (0.850–1.164)
Pregnancy > 41 weeks^b^	**1.411 (1.201–1.658)**	**1.492 (1.194–1.865)**	**1.323 (1.046–1.674)**
Labour induction^b^	1.011 (0.942–1.084)	0.964 (0.878–1.059)	1.066 (0.958–1.185)
Caesarean section^b^	0.995 (0.931–1.063)	0.933 (0.852–1.022)	1.063 (0.965–1.171)
Manual removal of placenta^b^	0.944 (0.848–1.052)	0.987 (0.854–1.140)	0.892 (0.759–1.049)
Postpartum haemorrhage^b^	**0.827 (0.703–0.973)**	0.816 (0.646–1.032)	0.840 (0.670–1.053)
Infant ≥ 4000g^b^	1.107 (0.908–1.267)	1.182 (0.946–1.476)	1.070 (0.904–1.266)
Apgar score < 7 at 5th min^b^	1.181 (0.861–1.620)	1.053 (0.698–1.592)	1.383 (0.846–2.261)
Perinatal death^b^	1.443 (0.987–2.108)	1.005 (0.574–1.760)	**2.123 (1.257–3.587)**
Stillbirth^b^	1.144 (0.696–1.881)	0.772 (0.372–1.602)	1.779 (0.891–3.549)
Neonatal death^b^	**2.154 (1.190–3.899)**	1.677 (0.693–4.056)	**2.796 (1.243–6.291)**

Significant differences shown in bold.

Results expressed in adjusted relative risk (aRR) and 95% confidence intervals (CI).

^a^Adjusted for age ≥ 35 years, body mass index ≥ 25 kg/m2, height ≤ 154 cm, infant gender = male, previous abortion history, any medical history.

^b^Adjusted for obstetric complications including hypertensive disorders and gestational diabetes in addition to^a^.

## Discussion

The results of this study revealed that in a city where universal childhood rubella immunization has been implemented for more than four decades, pregnant women found to be rubella seronegative at routine antenatal screening exhibited slightly but significantly difference characteristics. Specifically, there were more multiparous gravidae, which was contrary to expectation in view of the local government policy of repeated vaccination for postnatal women seronegative for rubella at routine antenatal screening. The slightly higher incidence of advanced age, short stature, and high BMI suggested that the different age distribution and anthropometric characteristics reflected underlying differences in health status, since women of both groups were from the same ethnic background, so that these women could have earlier waning of rubella immunity, or had a higher proportion of vaccine non-responders. Furthermore, there were significant differences in their past history and pregnancy outcomes, including both increased as well as reduced incidence of some specific outcomes. When analysis was stratified by parity status, nulliparous gravidae had increased post-dated pregnancy but reduced GH and GDM, and no increased adverse perinatal outcome. In contrast, multiparous gravidae, who should be at lower risk of adverse pregnancy outcomes^30^, actually had increased PE, post-dated pregnancies, neonatal death and total perinatal mortality. Therefore, our results indicated that rubella seronegative status in our multiparous gravidae plays proxy for subtle alteration in maternal immune response which increases occurrence of PE. Both increased PE and post-dated pregnancy had probably contributed to the increased perinatal and neonatal deaths, despite our policy of labour induction before 42 weeks gestation, as a gestation extending to 41 weeks and beyond is by itself associated with increased foetal hypoxia, antepartum and intrapartum foetal distress and acidosis, and intrauterine, neonatal and total perinatal deaths^31–33^, even though rubella sero-negativity has not been identified as a risk factor for prolonged and post-dated pregnancy^34,35^.

Our findings were at variance with the aforementioned case–control study that reported no effect of rubella sero-negativity on pregnancy outcome^11^ and pointed to the need for an adequate sample size in order to confirm or exclude an association with adverse pregnancy outcomes which may not be too frequent. The strengths of our study are the large size of the cohort which was ethnically homogenous, the uniform approach in clinical management and data collection for the generation of annual audits and statistical reports the accuracy of which has been validated previously^10^, the routine screening for rubella serology by accredited central laboratories using standard protocols and tests, and all being under a diligently carried out health policy of universal plus catch-up and remedial immunization available to all local citizens. Thus the data we utilized would be robust. The limitations of this study are the lack of detailed information on the individual cases with adverse outcomes, especially the cases of perinatal death. As well, no data on postnatal rubella vaccination was available from DH, but since their practice was not to follow-up these women for their vaccination response, even if such data were to be available, we would still have no clue as to whether these gravidae were vaccine non-responders or that they merely had waning of their antibody levels. However, once such associations have been identified, further studies can then be conducted to elucidate the cause of stillbirth, neonatal and total perinatal deaths among rubella seronegative gravidae.

Parity status is an important determinant of pregnancy outcome^30^, and multiparous status can reduce the adverse obstetric effects of demographic risk factors such as advanced maternal age^18,19^, and which could also modify the effect of maternal age on very preterm birth in twin pregnancies^36^. Furthermore, parity status is a major determinant of perinatal mortality, interacting with other factors such as foetal growth restriction and advanced maternal age, and increased perinatal mortality^37^ and stillbirth^38^ The unexpected association between multiparous status with adverse pregnancy outcomes found among rubella seronegative gravidae in this study probably reflects different maternal immunological and cytokine profiles. Among vaccine responders, rubella vaccination induces a marked increase in IL-4 and IL-10 levels^39^. One of the immunological features of PE is reduced in-vitro IL-10 secretion from peripheral blood mononuclear cells (PBMC)^40^ and IL-4 deficiency was shown to induce mild PE in mice^41^. Furthermore, the immunologic maladaptation associated with the development of PE includes a change in the circulating cytokines from Th2 to predominantly Th1 profile and a down-regulation of the immune regulatory system^42–46^. It is therefore possible that different cytokine profiles and altered Th1/Th2 response in rubella seronegative multiparous gravidae predispose them towards PE, which is a known cause of stillbirth^47^, the risk of which remained significantly higher compared with non-PE pregnancies despite improvement in perinatal care over the years^48^. As well, the risk of stillbirth is gestational age-related, being similar between expectant management and delivery at 38 weeks gestation, but then increases progressively so that by 41 weeks, it was 17.6 compared with 10.8 per 10,000 on-going pregnancies^49^. The explanation of the increased perinatal loss among rubella seronegative multiparous gravidae in this study remains to be determined. The likely contributing factors included the increased PE and tendency towards post-dated pregnancies. Further prospective studies are warranted to elucidate this association.

In conclusion, our study demonstrated for the first time that rubella seronegative status interacts with parity status to impact obstetric outcome. Nulliparous gravidae have reduced GDM and GH, but multiparous gravidae have increased PE, and neonatal and perinatal death, while there is increased post-dated pregnancy irrespective of parity status. Among pregnant women brought up under the cover of universal childhood rubella immunization, rubella seronegative status in the multiparous gravidae should be regarded as a risk factor for PE and perinatal mortality, and further studies are warranted to elucidate the underlying mechanisms and factors involved in this association.

## Data Availability

The data is the property of the hospital and the Hospital Authority, request for access should be made to the hospital through our department.
